# Hypertension pharmacogenetics and limitations in Africa – a focus on the *ACE*, *AGTR1* and *CYP2C9* genes

**DOI:** 10.1038/s41371-026-01121-0

**Published:** 2026-02-21

**Authors:** Rejoice T. Gomera, Wesley van Hougenhouck-Tulleken, Sarel J. Brand, Chantal van Niekerk, Kim Outhoff

**Affiliations:** 1 Department of Pharmacology, School of Medicine, Faculty of Health Sciences, University of Pretoria, 0028 Pretoria, South Africa; 2 Department of Nephrology, Dr George Mukhari Academic Hospital, Sefako Makgatho University, 0204 Pretoria, South Africa; 3 Centre of Excellence for Pharmaceutical Sciences, Department of Pharmacology, North- West University, Potchefstroom, 2520 South Africa; 4 Department of Chemical Pathology, School of Medicine, Faculty of Health Sciences, University of Pretoria, 0028 Pretoria, South Africa; 5 Department of Chemical Pathology, Tshwane Academic Division, National Health Laboratory Service, 0002 Pretoria, South Africa

**Keywords:** Genetics research, Genetics

## Abstract

Hypertension affects approximately a billion people worldwide and is a major risk for adverse cardiovascular and renal outcomes, particularly in the sub-Saharan African population. Only a small number of treated hypertensive patients achieve blood pressure control. Apart from factors such as poor medication adherence, the limited efficacy of some therapies could be attributed to inter-individual genetic variability. Thus, identifying genetic markers linked to antihypertensive drug response could assist in individualizing hypertension treatment and optimizing antihypertensive regimens to provide the greatest efficacy with the lowest risk for adverse effects. The Angiotensin-converting enzyme (*ACE*), Angiotensin II type I receptor (*AGTR1*) and Cytochrome P450 family 2 subtype C member 9 (*CYP2C9*) genes play pivotal roles in hypertension, and several key single-nucleotide variations (SNV) in these genes are known to have substantial effects on drug response in non-African populations. Numerous research findings corroborate that genotype-targeted antihypertension treatment regimens are more successful and can reduce costs by mitigating the likelihood of serious side effects. However, these findings may not be directly applicable to African populations due to the limited number of studies conducted and increased genomic variability within African populations. Two interconnected but distinct challenges impede translation of these benefits to African populations, namely limited implementation of pharmacogenetic testing for actionable drug-gene pairs across African healthcare systems, and the underrepresentation of African genetic ancestry in global genomic datasets, which hinders the identification and validation of population-specific variants. This review explores these dual challenges by examining the pharmacogenetic landscape of hypertension, with a focus on three clinically relevant genes: *ACE, AGTR1*, and *CYP2C9*. We highlight known gene-drug interactions, population-specific data gaps, and the need for research and infrastructure development to advance precision medicine in Africa.

## Introduction

Hypertension is a leading cause of death globally and causes a significant health burden in sub-Saharan Africa [1]. The consequences of hypertension are compounded by other burgeoning health risk factors such as obesity, diabetes mellitus, human immunodeficiency virus (HIV) infection and tobacco use, all of which increase the odds of myocardial infarction, stroke and kidney failure [2]. Chronic hypertension, which causes arteriolar endothelial damage, is recognized as the most common modifiable risk factor for adverse cardiovascular and renal events [3]. There are approximately 1.28 billion hypertensive adults aged 30-79 years globally, two-thirds of whom live in low- and middle-income countries [4, 5] (LMIC) where hypertension prevalence continues to grow [4]. The African region carries the highest burden of hypertension compared to the rest of the world, with a prevalence of 27%, compared to the North American region with the lowest prevalence of 18% [5].

Despite pharmacological interventions, hypertension remains poorly controlled. Blood pressure control rates are low globally, including in African countries [6], at least partially due to treatment resistance and low adherence resulting from multi-drug regimens and adverse reactions to active ingredients [7, 8]. Of note, eight African countries are included on the WHO list of the top ten countries with low hypertension treatment rates, ranging from 10-21% in both men and women (Table 1) [4].

**Table 1:** Top 10 countries with low hypertension treatment rates as reported by the World Health Organization (WHO) by sex.

Ranking	Sex	Country	Rate as % of all males/females with hypertension
1	Female	Rwanda	11
	Male	Rwanda	10
2	Female	Niger	15
	Male	Kenya	10
3	Female	Kiribati	15
	Male	Mozambique	10
4	Female	Ethiopia	16
	Male	Vanuatu	11
5	Female	Vanuatu	16
	Male	Solomon Islands	11
6	Female	Tanzania	17
	Male	Niger	12
7	Female	Solomon Island	17
	Male	Madagascar	13
8	Female	Madagascar	19
	Male	Uganda	13
9	Female	Mozambique	19
	Male	Togo	14
10	Female	Kenya	21
	Male	Burkina Faso	14

While pharmacological treatment has long been the mainstay of hypertension management, clinical responses vary widely between individuals. Increasingly, this variability is attributed to pharmacogenetic differences, i.e., genetic variations that influence drug metabolism, transport and target receptor sensitivity [7–10]. However, in African populations, two significant barriers impede the development and application of precision antihypertensive strategies. Firstly, few African countries have implemented pharmacogenetic testing in clinical settings, even when internationally recognized actionable gene-drug pairs are available [11]. Secondly, the majority of genome-wide association studies and pharmacogenomic databases are based on non-African populations, limiting our understanding of alleles and variants that may be highly relevant or even unique to African ancestry. These factors highlight the need for a deeper understanding of pharmacogenetics in African populations, where distinctive genetic variations may play pivotal roles in the efficacy and safety of antihypertensive therapies [10, 12, 13]. Addressing these genetic aspects could lead to more personalized and effective treatment strategies for hypertension in sub-Saharan Africa.

## The role of hypertension in chronic kidney disease

Hypertension and chronic kidney disease (CKD) are closely interlinked pathophysiological states; sustained hypertension worsens kidney function, while progressive renal decline can cause or exacerbate hypertension [14]. The pathophysiology of hypertension in CKD is complicated and involves the interaction of multiple factors, including a reduced number of functioning nephrons, sodium retention with volume expansion, diminished juxta-glomerular apparatus signalling and upregulation of the renin-angiotensin-aldosterone system (RAAS) [15]. The likelihood of developing hypertension increases as renal function declines, and hypertension incidence ranges from 60 to 90% in CKD patients, depending on the stage and underlying cause of their diagnosis [3, 14]. CKD contributes immensely to the global health burden as both its prevalence and the number of CKD-related deaths continue to rise [16]. In South Africa, CKD affects 10–15% of the population [5]. In the United States Renal Data System 2022 Annual Report, South Africa had the lowest incidence of treated end-stage renal disease (ESRD) in the world, at 11 per million population. However, South Africa was the only African region reported, and this likely reflects the limited availability of ESRD treatments as well as under-reporting in the African setting [2].

## he impact of HIV on hypertension

An estimated 39 million people live with HIV worldwide, of whom 25.6 million are in Africa [17]. The reported prevalence of hypertension in HIV-infected individuals varies between 9.8 and 28.7% in sub-Saharan African countries, [18–21] depending on the study design and sample size [18, 21]. Many HIV-infected individuals are unaware of their hypertension status, which may ultimately result in unintended cardiovascular and kidney disease, as hypertension may worsen pre-existing atherosclerosis [4, 21]. Additionally, variations in the *APOL1* gene, which encodes the apolipoprotein L1 protein, confer increased risks of kidney disease and are strongly linked to HIV-associated nephropathy [22]. *APOL1* high-risk variants are almost exclusively found in individuals of African ancestry, and variants of this gene increase the risk of many different types of kidney disease in African individuals [23].

## Hypertension treatment regimes

Individuals with hypertension who develop CKD are commonly treated with RAAS-inhibiting angiotensin-converting enzyme inhibitors (ACEi) or angiotensin II receptor blockers (ARBs) [24]. ACEi and ARBs are recommended first-line treatments in most clinical practice guidelines, including South Africa, due to their effectiveness in decreasing proteinuria, delaying the progression of CKD and delaying cardiovascular disease [25–27]. ACEi are competitive inhibitors of ACE and therefore prevent the conversion of angiotensin I to the powerful vasoconstrictor, angiotensin II, by removing a carboxy-terminal dipeptide [28]. ACEi are converted into their active metabolites by hepatic esterolysis using carboxylesterases or UDP-UDP-glucuronosyltransferase enzymes [29]. Further downstream, ARBs competitively block the angiotensin II type I receptors (AGTR1) and thus inhibit the effects of angiotensin II [30]. Candesartan and losartan are ARB prodrugs and are mainly metabolized by CYP2C9 enzymes (encoded by the *CYP2C9* gene), and transformed into active form of the drug [28].

Besides reducing angiotensin II-mediated peripheral arteriolar resistance, RAAS suppression by ACEi and ARBs also limits aldosterone secretion, reducing sodium and water retention, as well as sympathetic outflow, thus reducing blood pressure in a multifactorial manner [30].

## Pharmacogenetics and hypertension: actionable genes

The pharmacogenetics of hypertension is a complex landscape in which genetic variations significantly influence drug metabolism and response patterns. Numerous genetic variations are implicated in antihypertensive drug metabolism and efficacy, though only a subset has achieved “actionable” status in clinical practice with sufficient clinical evidence to influence or guide treatment decisions, such as adjusting drug choice, dosage or monitoring strategies.

The cytochrome P450 enzyme family plays a central role in metabolizing many drugs, including antihypertensive medications. For instance, CYP2C9 affects the metabolism of drugs such as losartan, potentially causing poor metabolizers to experience reduced efficacy due to decreased conversion to the active metabolite [31]. Conversely, extensive metabolizers may respond better to this ARB. Similarly, the clearance of other ARBs and calcium channel blockers may be affected by genetic variations of CYP3A4 and CYP3A5 enzymes [32]. Besides genes related to drug metabolism, receptor and transporter genes also impact drug response. Notably, *ACE* gene variations have been associated with differential responses to ACEi, while *AGTR1* variations directly affect the efficacy of ARBs. This review focuses on variations in these three genes that affect clinical response.

## Variations in the *ACE* gene affecting the clinical response to ACEi

The *ACE* gene plays a key role in the RAAS and the development of hypertension, as ACE converts angiotensin I to angiotensin II. Hyperactivation of the *ACE* gene increases transcription of the enzyme, ultimately leading to increased circulating angiotensin II, vasoconstriction, and elevated blood pressure [29]. Therefore, the *ACE* gene is an attractive candidate for studying genetic variants potentially associated with diminished responses to RAAS inhibitors, such as ACEi and ARBs. Indeed, genetic variations in the *ACE* gene, a 21 kb gene consisting of 26 exons [33] located on chromosome 17q23, are known to be associated with diminished effects of ACEi in many, mostly non-African populations [5].

There are more than 100 *ACE* gene variations listed in the National Centre of Biotechnology Information (NCBI) records, most of which are SNVs [34]. The most studied and significant SNV is rs1799752, which is an insertion (I)/deletion (D) in intron 16. Various reports have indicated that the D allele [35, 36] of this gene is associated with a superior antihypertensive response, in contrast to others who ascribe these superior effects to the I allele [37, 38].

The rs1799752 variation has been studied in two African countries, namely Egypt and Burkina Faso. Three of four Egyptian studies found an association between this SNV and hypertension [39, 40], while the other found none [41]. In Burkina Faso, an association was also demonstrated between rs1799752 and hypertension [42]. A SNV located in close proximity to rs1799752 is rs4343. This variant demonstrates complete linkage disequilibrium with rs1799752, as the two alleles occur together more often than expected. When the two biallelic *ACE* variants, rs1799752 (I/D) and rs4343 (G/A), are analysed together, four theoretical haplotype combinations may occur on a single chromosome, namely I–G, I–A, D–G, and D–A. The rs4343 A allele has been observed to co-segregate with the insertion allele, while the G allele co-segregates with the deletion allele of rs1799752. In a Genome-Wide Association Study (GWAS) of hypertensive participants, the rs4343 variant, present in 16.2% of the 1023 participants, correlated with diminished ACEi activity [43]. Importantly, hypertensive patients carrying the variation demonstrated no improvement in blood pressure readings after a year of follow-up, despite being treated with ACEi [43] as the SNV alters regulatory motifs for several transcription factors that affect the metabolite levels [34, 44–46].

Other commonly studied and significant *ACE* SNVs include rs1800764, rs4329, rs4340, rs4316, rs4344 and rs4291 [34, 44–46] (see Table 2). Variants rs4329, rs4316 and rs4344 have been hypothesised to limit the effectiveness of ACEi as they affect the translation of ACE. Individuals carrying these variants are less responsive to ACEi therapy compared to patients without the variant [40, 47]. Table 2 highlights the biogeographical distribution of allele frequencies for select genetic variations of several populations, including five African, as reported by the 1000 Genomes Project, and their probable clinical significance.

**Table 2:** Biogeographical distribution of allele frequencies of ACE variations and probable clinical significance.

Variation	Allele	ACB	ASW	ESN	GWD	LWK	MSL	YRI	AFR total	AMR total	EAS total	EUR total	SAS total
rs4343 (increases ACE activity [43])	A/G*	0.28	0.20	0.27	0.15	0.10	0.17	0.21	0.20	0.39	0.32	0.56	0.37
rs1800764 (increases ACE activity [48])	C/T*	0.15	0.21	0.08	0.06	0.13	0.05	0.06	0.10	0.63	0.64	0.54	0.60
rs4329 (increases ACE activity [47])	G/A*	0.58	0.55	0.61	0.60	0.56	0.61	0.59	0.59	0.43	0.33	0.58	0.38
rs4316 (moderate to high linkage disequilibrium marker for developing hypertension [49])	C/T*	0.39	0.40	0.35	0.36	0.42	0.35	0.36	0.38	0.56	0.67	0.42	0.62
rs4344 (increases ACE activity [47])	G/A*	0.41	0.43	0.37	0,38	0.45	0.38	0.38	0.40	0.57	0.67	0.43	0.62
rs4291 (increases ACE activity [48])	A/T*	0.36	0.30	0.32	0.42	0.30	0.37	0.38	0.35	0.28	0.35	0.37	0.36

*allele = variant allele.

*RsID* unique identifier for the genetic variation assigned by the database, *ACB* african caribbean in Barbados, *ASW* african ancestry in southwest US, *ESN* esan in Nigeria, *GWD* gambian in the western division, *LWK* luhya in webuye, Kenya, *MSL* mende in sierra leone, *YRI* yoruba in ibadan, Nigeria, *AFR* Africa, *AMR* America, *EAS* East Asia, *EUR* European, *SAS* South Asia.

Although ACE variants have been extensively studied in non-African populations, data from African countries remain limited. Data from the 1000 Genomes Project were derived from only five African populations and two African-ancestry populations. Africa has a very high genetic diversity within and between populations, which is important to note. Data from, for example, African Americans, people from Kenya, and participants from Egypt will differ considerably in the genetic variations present in the particular study population yet fewer studies, usually with small sample sizes, have been conducted among African populations compared to the global north [48].

In addition, clinical pharmacogenetic testing for *ACE*-related polymorphisms is not routinely available in most African healthcare systems. This lack of infrastructure hampers the integration of genotype-guided prescribing into clinical practice and underscores the need for broader implementation efforts and African-led research to assess the relevance of these variants across the continent’s diverse populations.

## Variations in the *AGTR1* gene affecting clinical response to ARBs

Most of the physiological effects of angiotensin II, such as vasoconstriction, aldosterone release and sodium reabsorption, are transduced through the Angiotensin II Type 1 receptor (AGTR1), a key RAAS protein which is encoded by the *AGTR1* gene, localized to chromosome 3q21-q25. The *AGTR1* gene exceeds 55 kb and consists of five exons and four introns [48]. ARBs competitively block AGTR1, which is predominantly present in vascular smooth muscle cells, to exert their antihypertensive effects [47].

Of note is the rs5186 variant, also known as A1166C, which is the best-studied *AGTR1* SNV. Individuals with the heterozygous AC genotype display a significant reduction in blood pressure in response to treatment [47, 49]. Conversely, carriers of the AA genotype have resistant hypertension despite compliance with antihypertensive treatment in African Americans and European Americans. Furthermore, individuals carrying the rs275651 and/or rs5186 variants have no significant response to RAAS-inhibiting treatment. In Egypt, a high frequency of this SNV was associated with hypertension [50]. Similar to a study in Asia [51], the African populations of Cameroon and Algeria, no association was observed between the rs5186 variant and hypertension [52] concluding that the rs5186 variant is not associated with the antihypertensive effects of RAAS blockade. Several other SNVs in *AGTR1*, including rs27651, rs422858, rs427653 and rs7808671 (Table 3) affect the ARB binding site. Functional analysis of these SNVs has shown altered binding affinities, cell surface expression, and response to ARBs [53]. The effects of *AGTR1* SNVs are not clear, as there are conflicting results among different populations. Table 3 shows *AGTR1* allele frequency patterns for specific variants, including the previously mentioned five West and East African populations, reported by the 1000 Genomes Project, and their probable clinical significance. Once again, the lack of robust data not only reduces our ability to understand population-specific genetic influences but also perpetuates disparities in precision medicine. As a result, opportunities to implement genotype-guided prescribing are missed, leaving treatment decisions reliant on generalized data that may not reflect our African genetic diversity.

**Table 3:** Biogeographical distribution of allele frequencies of *AGRT1* variations and probable clinical significance.

Variation	Allele	ACB	ASW	ESN	GWD	LWK	MSL	YRI	AFR total	AMR total	EAS total	EUR total	SAS total
rs5186 (Affect mRNA stability [54])	A/C*	0.03	0.09	0.01	0.01	0.01	0.01	0.01	0.02	0.23	0.06	0.27	0.07
rs275651 (Decreases binding affinity [53])	A/T*	0.75	0.74	0.69	0.64	0.64	0.65	0.77	0.70	0.88	0.87	0.83	0.88
rs422858 (Decreases binding affinity [53])	A/C*	0.24	0.25	0.29	0.35	0.35	0.31	0.23	0.29	0.12	0.11	0.17	0.19
rs27651 (Decreases binding affinity [53])	T/C*	0	0	0	0.02	0	0.02	0	0.01	0	0	0	0
rs78086717 (Decreases binding affinity [53])	A/G*	0.08	0.06	0.05	0.07	0.06	0.04	0.08	0.06	0.01	0	0	0

*allele = variant allele.

RsID unique identifier for the genetic variation assigned by the database, *ACB* African Caribbean in Barbados, *ASW* African Ancestry in Southwest US, *ESN* Esan in Nigeria, *GWD* Gambian in the Western Division, *LWK* Luhya in Webuye, Kenya, *MSL* Mende in Sierra Leone, *YRI* Yoruba in Ibadan, Nigeria, *AFR* Africa, *AMR* America, *EAS* East Asia, *EUR* European, *SAS* South Asia.

## Variations in the *CYP2C9* gene affecting ARB metabolism

The Cytochrome P450 (CYP450) iso-enzyme proteins are monooxygenases that catalyse many reactions involved in drug metabolism and the synthesis of cholesterol, steroids, and other lipids. The enzymes are mainly localized in the liver, although they may also be found in the intestine, and are subject to inter-individual variation. The *CYP2C9* gene, which encodes one subtype, is located on chromosome 10q23.33 and consists of nine exons [54]. This gene is highly polymorphic, including functional variants that are of pharmacogenetic importance. Altered drug metabolism caused by the variations plays a major role in the pathogenesis of adverse drug reactions [55]. Of note is that ARBs are chiefly metabolized by CYP2C9 enzymes.

Genetic variations in drug-metabolizing enzyme activity can lead to wide interindividual variability in drug response. Individuals treated with the same dose may display altered efficacy or even toxicity based on their metabolic profile [56], that includes four possible categories: poor metabolizer, intermediate metabolizer, extensive metabolizer, or ultra-extensive metabolizer. Poor metabolizers may have an accumulation of the parent drug and thus an increased risk of acute kidney injury, which may cause further damage to those with pre-existing renal impairment. Additionally, a longer half-life may lead to sustained antihypertensive as well as toxic effects. On the other hand, extensive metabolizers may experience reduced efficacy due to increased clearance, necessitating more frequent dosing. For ARB prodrugs such as losartan, candesartan and olmesartan that rely on enzymatic conversion to the active metabolite, the opposite is true.

Functional evidence of altered activity has been reported for at least 20 SNVs in *CYP2C9* [57]. The most common coding variations in *CYP2C9* are CYP2C9*2 and CYP2C9*3, which differ from the most common genotype CYP2C9*1. These pathogenic variations have been extensively studied in European and Asian populations, but there is limited knowledge of their frequency in African populations [57, 58]. According to the PharmaGKB database, CYP2C9*2 (rs1799853) and CYP2C9*3 (rs1057910) (Table 4) are tier 1 variants as substantial evidence supports their importance in pharmacogenomics [33]. Studies have demonstrated that individuals with the CYP2C9*2 variant display approximately a 50% reduction in drug metabolism and clearance rates compared to those with the most common genotype, CYP2C9*1. CYP2C9*2 and CYP2C9*3 result in significantly reduced enzyme activity, which may lead to poor metabolism [59]. In a study of European hypertensive patients with CKD, 16.7% of the study population carried the CYP2C9*3 variant. No changes in blood pressure were observed in patients with CYP2C9*3, which impaired CYP2C9 activity [59, 60]. Attenuated clinical effects of ARBs were observed in CKD patients with the CYP2C9*2 and CYP2C9*3 variants compared with individuals with the CYP2C9*1 genotype, who showed a reduction in both systolic and diastolic blood pressure [61]. Contrary to these findings, similar reductions in both systolic and diastolic blood pressure among carriers of the CYP2C9*2 variants compared with carriers of the CYP2C9*1 genotype have been observed in an Asian population [61]. These results highlight the need to fully understand the role of the *CYP2C9* genotype in clinical outcomes [54]. The limited evidence from African cohorts remains sparse and inconclusive, leaving a blind spot in global pharmacogenomics (Table 4). This shortfall leads to underrepresentation in GWAS and impedes clinical translation. It also risks widening health inequities, as treatment strategies continue to rely on data that only partially reflects African diversity. This limits the development of inclusive, evidence-based strategies for managing hypertension, especially with respect to clinically actionable drug-gene pairs.

**Table 4:** Biogeographical distribution of allele frequencies of CYP2C9 variations and the probable clinical significance.

Variation	Allele	ACB	ASW	ESN	GWD	LWK	MSL	YRI	AFR total	AMR total	EAS total	EUR total	SAS total
rs1799853 (decreases metabolism of ARBs [31])	C/T*	0.03	0.04	0	0	0	0	0	0.01	0.10	0	0.12	0.03
rs1057910 (decreases metabolism of ARBs [75])	A/C*	0.01	0.02	0	0	0	0	0	0	0.04	0.03	0.07	0.11

*allele = variant allele.

*RsID* unique identifier for the genetic variation assigned by the database, *ACB* African Caribbean in Barbados, *ASW* African Ancestry in Southwest US, *ESN* Esan in Nigeria, *GWD* Gambian in the Western Division, *LWK* Luhya in Webuye, Kenya, *MSL* Mende in Sierra Leone, *YRI* Yoruba in Ibadan, Nigeria, *AFR* Africa, *AMR* America, *EAS* East Asia, *EUR* European, *SAS* South Asia.

## Limitations in African pharmacogenetics: implementation and genomic representation gaps

There are several challenges limiting the use of pharmacogenetic approaches in African contexts. Firstly, pharmacogenetic testing remains uncommon, particularly outside of research settings. In many LMICs, public health services are hesitant to adopt precision medicine, as the high upfront costs place it low on their list of priorities. Moreover, implementing testing in the absence of a robust evidence base to guide clinical practice offers little utility and may even be considered unjustifiable. However, pharmacogenomic studies in native African populations are scarce due to inadequately equipped research facilities, possible inertia, and limited funding and local expertise [62, 63]. Although middle-income countries such as South Africa are advancing in genomic technologies, this is not the case for most of Africa, which lags behind the USA, Europe and Asia [64]. These results may be indicative of problems in African healthcare systems, such as a lack of education and inadequate therapy that result in suboptimal blood pressure control [63], but pharmacogenetic differences also likely play a role in these patients. Some developed countries, such as Canada and Australia, have implemented pharmacogenetic testing in clinical practice, which has ultimately reduced the medical cost for their hypertensive patients [65]. In Africa, hypertension still has a significant economic cost. Reports from South Africa, Kenya, and Ethiopia indicate that the treatment cost of hypertension per patient is approximately US$125, US$305 and US$92, respectively, per year [63, 65–67]. Given that most African countries are LMICs and that hypertension, including its adverse effects, is a significant health burden, pharmacogenetic testing could significantly reduce treatment costs. Lack of funding and infrastructure for routine pharmacogenomic testing impacts the feasibility of such approaches [63]. Although sufficient evidence may not exist to justify the publication of guidelines, investing in infrastructure and skills development represents an important step toward change.

A practical way forward would be to adopt a stepwise approach that leverages existing infrastructure and affordable methods, rather than replicating resource-intensive models from high-income countries. This could involve prioritising a small number of well-established, clinically relevant drug-gene pairs for hypertension, developing simplified genotyping platforms or point-of-care assays for these variants, and embedding them into clinical decision support tools. At the same time, establishing local biobanks and fostering regional collaborations would support the gradual generation and validation of data across diverse African populations. Such an approach balances feasibility with clinical value, reduces reliance on trial-and-error prescribing, and builds the evidence base needed to demonstrate the importance of pharmacogenetics in settings where it may currently be viewed as a lower priority [68, 69]. The required infrastructure extends beyond mere laboratory capacity, encompassing biobanking facilities, bioinformatics expertise, and training programs that empower African scientists to lead this research.

Secondly, Africa continues to be underrepresented in global genomic research, despite the fact that contemporary diagnosis, prognosis and therapy depend heavily on robust clinical evidence. Due to the lack of data in Africa, extrapolations to Africans often have been made from other ethnic groups, typically the African-American population. However, these extrapolations are unlikely to be accurate due to non-African admixture in the African-American population, the relatively limited geographical area from which African-Americans originated i.e., Western Central Africa [63], and the enormous genetic diversity of African populations that did not contribute to the African-American population [63, 70–74] Even when studies are available, they typically cover only a narrow range of African populations. For instance, the 1000 Genomes Project includes data from just five regions across the continent, offering only a glimpse of Africa’s vast genetic diversity. Consequently, many potentially important genetic variants remain either undiscovered or insufficiently validated. Encouragingly, initiatives such as the Human Health and Heredity in Africa (H3Africa) and the Southern African Human Genome Programme (SAHGP) have begun to address this gap. These programmes are working to characterize the African genome and establish baseline frequencies for known genetic variants, providing a much-needed foundation for future pharmacogenetic research and more inclusive clinical translation [68, 69].

By enabling more research on African populations, a deeper understanding of allele frequencies may be unlocked, which is an essential foundation for developing and publishing comprehensive pharmacogenetic testing guidelines that accurately predict drug response, minimize adverse effects, and optimize therapeutic outcomes. Conceptually, hypertension pharmacogenomic studies aim to enable the utilisation of genetic information. This occurs in concert with pertinent clinical or demographic data to select individualised antihypertensive regimens likely to provide the greatest efficacy with the lowest risk of adverse effects. As such, pharmacogenomics in hypertension holds the promise of identifying genetic biomarkers for antihypertensive drug response, which should ultimately facilitate improved treatment selection. Research in the field is also likely to enhance our understanding of hypertension and the mechanisms by which the various drugs produce their effects. Given that hypertension is a modifiable risk factor, more research in this area can help identify crucial pharmacogenes and variants, which may improve the quality of healthcare in African patients.

## Conclusion

Hypertension continues to impose a disproportionate burden in Africa, where trial-and-error prescribing is the norm. Two critical barriers must be addressed to advance equitable precision medicine: the minimal integration of pharmacogenetic testing into clinical care, and the persistent underrepresentation of African populations in genomic research. Strengthening the implementation of existing actionable drug–gene pairs and simultaneously expanding locally driven genomic studies will be essential to ensure that treatment strategies reflect the continent’s unique genetic diversity. By closing both the implementation and discovery gaps, Africa can move towards more effective, inclusive and sustainable approaches to hypertension management.
